# Raman spectroscopy combined with machine learning algorithms to detect adulterated Suichang native honey

**DOI:** 10.1038/s41598-022-07222-3

**Published:** 2022-03-02

**Authors:** Shuhan Hu, Hongyi Li, Chen Chen, Cheng Chen, Deyi Zhao, Bingyu Dong, Xiaoyi Lv, Kai Zhang, Yi Xie

**Affiliations:** 1 College of Software, Xinjiang University, Ürümqi, 830046 China; 2 College of Information Science and Engineering, Xinjiang University, Ürümqi, 830046 China; 3 Guangzhou Panyu Polytechnic, No. 1342 Shiliang Road, Guangzhou Panyu, 511483 Guangdong China; 4 Xinjiang Aiqiside Testing Technology Co., Ltd., Ürümqi, 830046 China

**Keywords:** Applied optics, Chemical engineering, Optics and photonics

## Abstract

Zhejiang Suichang native honey, which is included in the list of China’s National Geographical Indication Agricultural Products Protection Project, is very popular. This study proposes a method of Raman spectroscopy combined with machine learning algorithms to accurately detect low-concentration adulterated Suichang native honey. In this study, the native honey collected by local beekeepers in Suichang was selected for adulteration detection. The spectral data was compressed by Savitzky–Golay smoothing and partial least squares (PLS) in sequence. The PLS features taken for further analysis were selected according to the contribution rate. In this study, three classification modeling methods including support vector machine, probabilistic neural network and convolutional neural network were adopted to correctly classify pure and adulterated honey samples. The total accuracy was 100%, 100% and 99.75% respectively. The research result shows that Raman spectroscopy combined with machine learning algorithms has great potential in accurately detecting adulteration of low-concentration honey.

## Introduction

China is not only a large beekeeping country, but also the largest honey exporter. Among the many honeys, Suichang native honey, a special honey from Lishui County, Zhejiang Province, China, is famous for its amber color and unique taste. Therefore, it has been shortlisted in China’s National Geographical Indication Agricultural Products Protection Project List in 2021. In recent years, the problem of honey adulteration has become more serious. It has seriously damaged the interests of both producers and consumers. Suichang native honey is also suffering from it. Therefore, it is urgent to develop an efficient and accurate honey counterfeit detection technology. Honey contains fructose, glucose, maltose, sucrose, etc. The raw materials of these carbohydrates are not only easy to obtain, but also adulterated with these carbohydrates, the taste of honey is significantly improved, and it is difficult to detect. These reasons have caused that honey has always been an easy target of adulterators for economic gains. Many researchers have utilized different analytical techniques to face this problem, such as high performance liquid chromatography, isotope analysis or near-infrared spectroscopy^1^. The limitations of liquid chromatography and isotope analysis are high analysis costs, high instrument maintenance costs, and cumbersome sample preparation operations^2,3^. But the Raman spectroscopy instrument is simple to operate and has low maintenance cost. And Raman spectroscopy is often used as a complementary approach to near-infrared spectroscopy^4^. Generally, substance molecules with high Raman intensity have low near-infrared intensity. This also shows that Raman spectroscopy can explore the molecular information in substances and analyze problems from different angles of near-infrared spectroscopy. Besides, Raman spectroscopy does not require preprocessing. The key advantages of using the Raman spectroscopy approach are high resolution and less overlapping bands^5^.

Raman spectroscopy is a scattering spectrum, which can reflect the group composition of the molecule through the Raman scattering produced by the vibration and stretching of various groups in the molecule. Raman spectroscopy is a common method in the food measurement field^6,7^. It has a wide range of applications in the detection and classification of honey adulteration. For example, in 2012, Shuifang Li et al. used Raman spectroscopy combined with partial least squares linear discriminant analysis (PLS-LDA) to detect the feasibility of beet syrup in honey. Study the nature of honey, and reach a positive conclusion^8^. In 2018, Oroian et al. used PLS-LDA to detect adulteration of fructose, glucose, sucrose, maltose, hydrolyzed inulin syrup in honey. And they observed a total accuracy of 96.54%^5^.

The Partial least square (PLS) method is a commonly used mathematical optimization technique. This method can eliminate the worthless spectral information in the original data, improve the modeling speed while retaining the original data to the maximum extent information contained in. Therefore, PLS is widely used in spectral analysis^9–11^.

The Probabilistic Neural Network (PNN) developed by Specht is a general classifier^12^ that solves the problem of pattern classification. The advantages of PNN are easy training and fast convergence speed. The hidden layer of PNN uses radial basis functions to make it have a high tolerance for samples. Therefore, it is often used in solving classification problems^13–15^.

Support vector machine (SVM) is one of the most versatile methods used to solve binary classification problems. SVM can efficiently solve classification and regression problems. Besides, SVM is based on support vectors, so it can obtain better classification results in few-shot learning. For these reasons, the Raman spectroscopy combined with SVM is widely used in biological classification^16–18^.

Convolutional neural network (CNN) is a feedforward neural network with convolution calculations. It is one of the representative algorithms of deep learning. Acquarelli et al. argues that CNN can be effectively applied to the classification of vibration spectrum data^19^. CNN shares a convolution kernel for different regions, which greatly reduces the required parameters while being robust. The generalization ability of CNN is also improved compared to traditional neural networks. Therefore, CNN combined with Raman spectroscopy is widely used in biological classification^20,21^.

The aim of this study is to propose a Raman spectroscopy combined with machine learning to identify honey adulteration. In the experiment, we compressed spectral data by Savitzky-Golay smoothing and PLS in sequence, and then input the PLS features into the CNN, SVM, and PNN models. Finally, we analyzed and compared the discrimination results. The result shows that the accuracy rate of SVM and CNN is 100.00%, and the accuracy rate of CNN is 99.75%. In this study, we verified the feasibility of the machine learning algorithm combined with Raman spectroscopy. This study is expected to improve the accuracy of low-concentration honey adulteration.

## Experimental materials

### Sample preparation

In this study, pure honey samples were collected by beekeepers in Lishui County, Zhejiang Province, China. The syrup was concentrated till 65° Brix syrup prepared from maltose purchased in the market. The adulterated sample was obtained by mixing 190 g of pure honey with 10 g of maltose syrup. We prepared 100 pure honey samples and 100 adulterated samples. All samples had Raman measurements within 24 h after preparation.

### Raman spectrum measurements

The Raman spectra was recorded using laser Raman spectroscopy (ACCUMAN SR-510 PRO, Ocean Optics) ranged from 200 to 4000 cm^−1^. In this study, we spread the sample on the surface of commercially available aluminum foil to form a film with a thickness of about 1 mm and an area of about 1 cm^2^. The light source was a 785 nm laser which was emitted by a laser transmitter equipped with the instrument. The laser power level was set to 7. The integration time was set to 30 S, and the average times was set to 2, the sampling interval was set to 5 S, and the times was set to 50. Most Raman spectrum has a lot of fluorescence noise. In this study, we used the fluorescence removal function of the AccumanRS software to perform fluorescence removal on the original spectrum. The parameter of fluorescence removal was set to 33. The software version number is 1.00.79. We recorded two spectra at different locations for each sample and took the mean spectrum for further analysis. We also show the spectra of five samples from both categories, pure honey and adulterated honey in the Supplementary file.

## Data processing

### Data preprocessing

**Figure 1:**
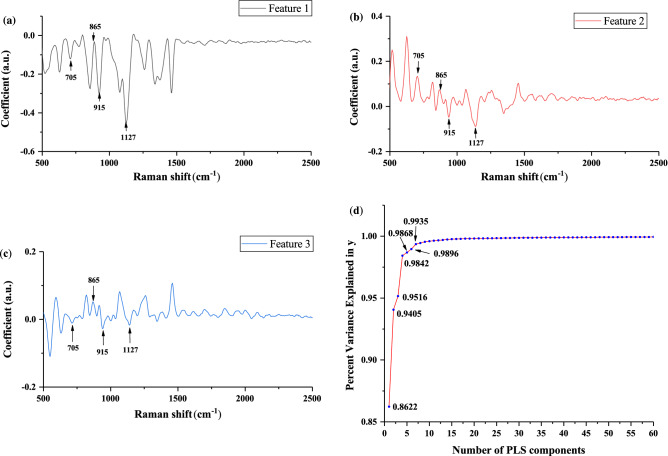
load curve of PLS first 3 features (**a**–**c**) and the feature cumulative variance explanation rate curve.

To better reduce the noise, we used Savitzky–Golay smoothing for the spectral data after fluorescence removal. The filter window size was set to 9. All honey samples were divided into training sets (80%, 160 samples) and test sets (20%, 40 samples). After that, we submitted the spectral data to the PLS program. Then we selected the appropriate number of PLS features by analyzing the cumulative variance explanation rate of the features^22^. These features were selected as the input to the classifier through tenfold cross validation. In Fig. 1a–c show the load curve of the first 3 features. The load graphs represent the most significant peak in each feature space. There are obvious intensity peaks at 705 cm^−1^, 865 cm^−1^, 915 cm^−1^, 1065 cm^−1^, 1127 cm^−1^, 1373 cm^−1^, and 1461 cm^−1^. It can indicate that the features do reflect the characteristics of the original data. Figure 1d shows the cumulative variance explanation rate of the first 7 features has reached 0.9935, and the variance explanation rate of a single feature after the seventh feature has been lower than 0.001. Thus, the first 7 features can fully express the features of the original data^23^. Therefore, the first 7 features of the PLS result are selected as the input of CNN, PNN, and SVM. The related program runs on python 3.6.

### Classification models

**Figure 2:**
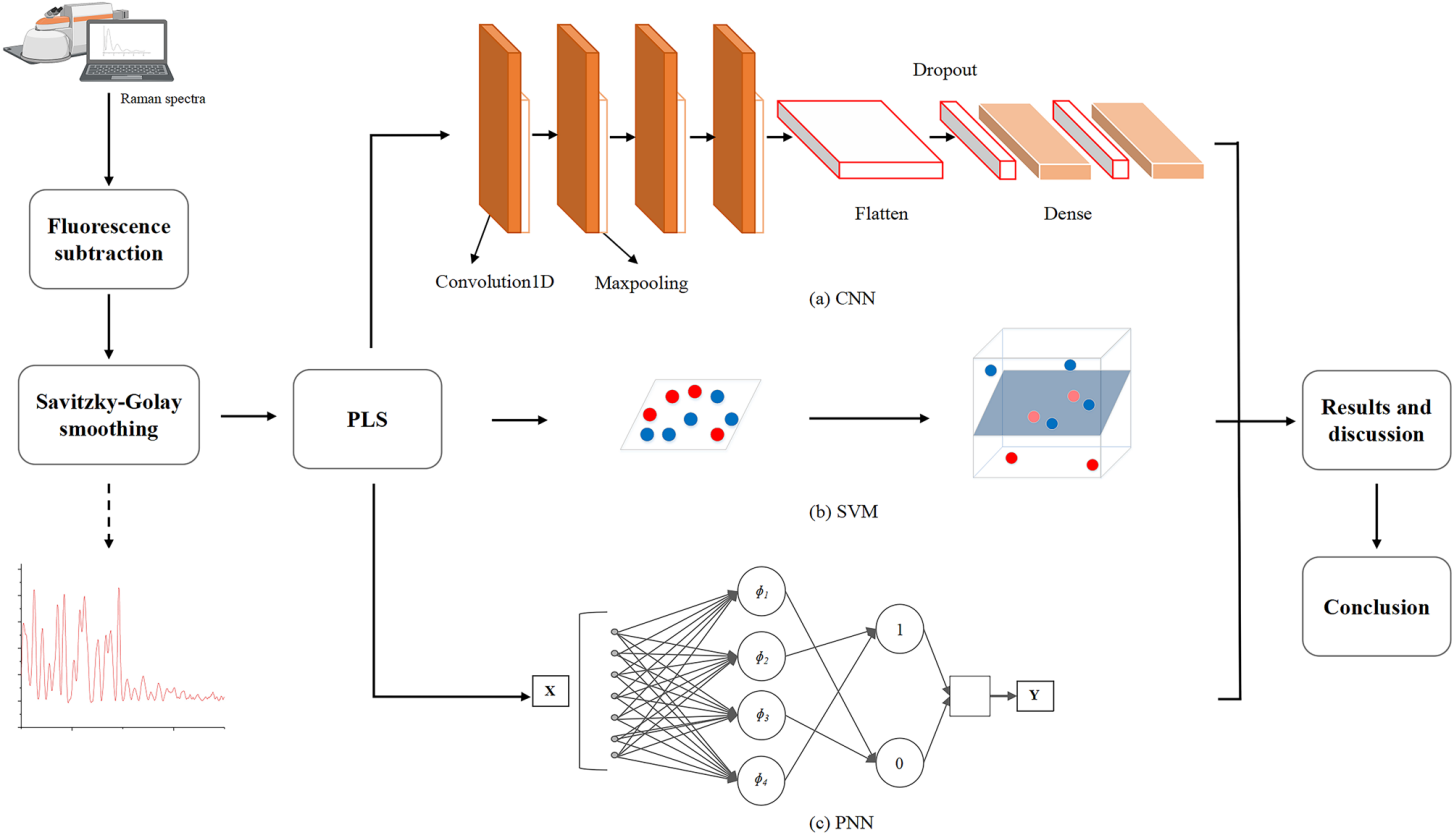
Experimental process.

Each of the first four layers of the CNN model consists of a convolution layer and a pooling layer. The pooling layers used maximum pooling, which avoids the fuzzification effect of average pooling. The first layer had 128 convolution kernels, and the size of convolution kernels was set to 8. The pooling size was set to 4, and the pooling step was set to 2. The second layer had 64 convolution kernels, and the size of convolution kernels was set to 3. The pooling size was set to 4, and the pooling step was set to 2. The third and fourth layers had 32 convolution kernels, and the size of convolution kernels was set to 2. The pooling size was set to 2, and the pooling step was set to 2. The fifth layer was the Flatten layer. The sixth and seventh layers were Dense layers, and the Dropout layer was inserted before the two Dense layers. In this study, the batch size of the CNN program was set to 32, the learning rate was set to 0.001, the epochs value was set to 200, and the loss function was “categorical_crossentropy”. The structure of CNN is shown in Fig. 2. The CNN program runs on python3.6.

The first layer of the PNN model was the input layer, the second layer was the radial base function (RBF) layer, the third layer was the summation layer, and the fourth layer was the input layer. The network spread value of the PNN program was 0.01. The PNN program runs on matlab2016a.

**Figure 3:**
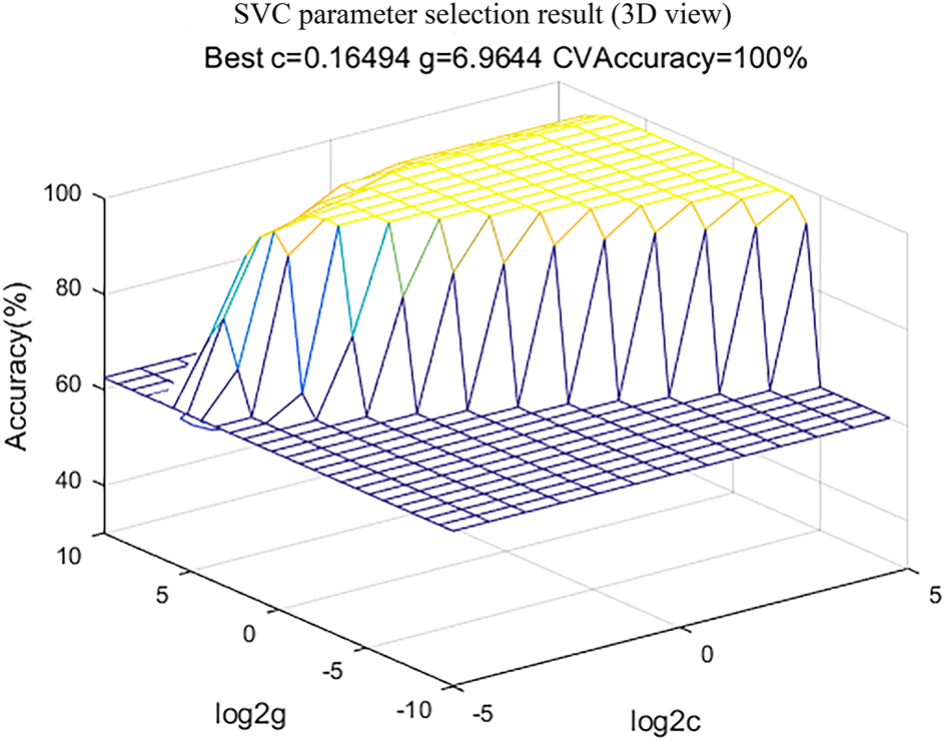
SVC parameter selection result (3D view).

SVM can map the data to the high-dimensional feature space through the kernel method to find the largest separation hyperplane. The kernel function was set to RBF. The key parameters of SVM are finding the optimal C and g parameters. This study used a grid method in a limited range to find them. The range of c was 2^−5^ up to 2^5^, and the range of g was 2^−10^up to 2^10^^24^, the step of the exponent part was all set to 1. The result of program SVC parameter selection is shown in Fig. 3, which shows the accuracy of different C and g in the process of grid search. The SVM program runs on matlab 2016a.

## Results

### Spectral analysis

**Figure 4:**
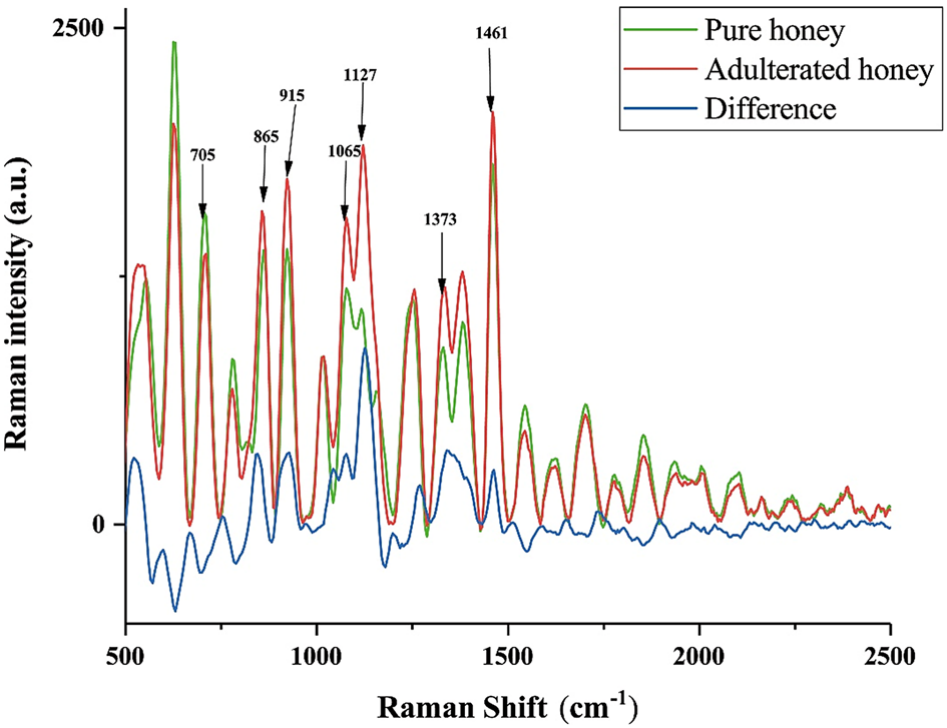
Mean spectra of samples.

Figure 4 presents the Raman spectra of Suichang native honey. The identification of honey adulteration mainly relies on carbohydrate spectra, the Raman spectra of non-carbohydrate substances are hidden in the fluorescent background, which is not conducive to classification^25^. We used the part between 500 and 2500 cm^−1^ for interpretation as only here the spectral information is related to carbohydrates. According to Fig. 4, in the region between 500 and 2500 cm^−1^, peaks were observed at 705 cm^−1^, 865 cm^−1^, 915 cm^−1^, 1065 cm^−1^, 1127 cm^−1^, 1373 cm^−1^ and 1461 cm^−1^. The spectral peak at 705 cm^−1^ of adulterated honey is lower than pure honey, while the remaining Raman peaks are higher than those of adulterated honey. The peak and corresponding substances are indicated in Table 1. The peak at 705 cm^−1^ is attributed to the C=O in the carbohydrate aldehyde group. The peaks at 865 cm^−1^, 915 cm^−1^, and 1376 cm^−1^ are attributed to the CH of organic matter in honey. And the peaks at 915 cm^−1^ and 1373 cm^−1^ are attributed to the hydroxyl groups in carbohydrate, and the characteristic peaks at 1127 cm^−1^ are attributed to the C-O bonds in carbohydrate and glycosidic bonds. The difference in the intensity of these characteristic peaks provides a key basis for the subsequent discrimination.

**Table 1:** Peak positions and assignments of main Raman bands^8^.

Wavenumber (cm^−1^)	Molecular information
705	C=O bond stretching vibration
865	C–H bond vibration
915	C–H and C–OH bond bending vibration
1127	C–O bond stretching vibration
1373	C–H and O–H bond bending vibration
1461	H–C–H bond bending vibration

### Model evaluation

This study evaluated models from specificity, sensitivity and accuracy. The equations of the three indicators are as follows:

1 $$Specificity = \frac{TN}{{TN + FP}}$$

2 $$Sensitiv{\text{ity}} = \frac{TP}{{TP + FN}}$$

3 $$Accuracy = \frac{TP + TN}{{TP + FP + FN + TN}}.$$

**Figure 5:**
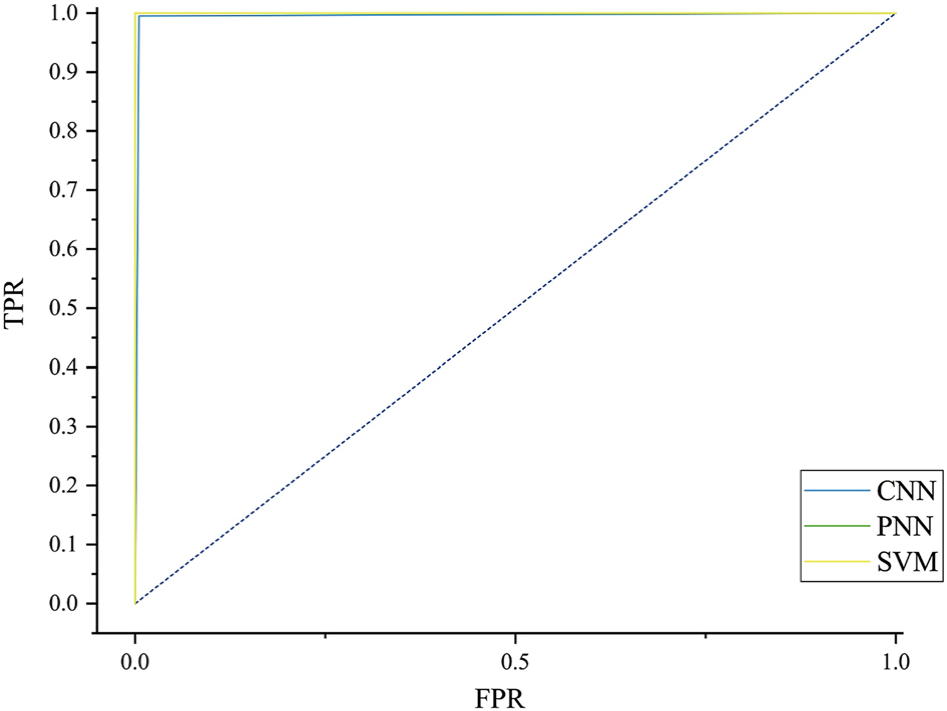
ROC curves of models.

TP, TN, FP, and FN correspond to true positive, true negative, false positive, and false negative, respectively. Table 2 shows the three indicators of each model. Figure 5 is the receiver operating characteristic curve (ROC curve). The area under curve (AUC) of ROC curve can show the classification ability of the classifier more intuitively. The AUC of SVM and PNN are both 1, and the AUC of CNN is 0.9975. All three classifiers show excellent classification capabilities on this problem. According to Table 2, the sensitivity, specificity, and accuracy of the PNN and SVM are 100%. The sensitivity of CNN is 99.49%, the specificity is 100%, and the accuracy is 99.75%.

**Table 2:** Model indicators.

Model	Sensitivity (%)	Specificity (%)	Accuracy (%)
CNN	99.49	100	99.75
PNN	100	100	100
SVM	100	100	100

## Discussion

It can be seen from Table 1 that the difference in characteristic peak intensity mainly comes from carbohydrates^8^. The peak at 705 cm^−1^ is related to fructose. Since fructose in pure honey generally accounts for about 50% of the sugar content, and the maltose is a disaccharide formed by the connection of two glucoses. Therefore, the concentration of fructose in adulterated honey is lower than that in pure honey. The increase in the intensity of other characteristic peaks may be related to the higher sugar content of maltose syrup than pure honey. CNN, PNN and SVM can still achieve a good discrimination effect even at a low concentration of 5% adulteration. Compared with PNN and SVM, the reason for the lower sensitivity and lower accuracy of CNN may be related to the less samples. Compared with PLS-LDA, these three models have further improved the classification accuracy. The possible reason is that the RBF layer in PNN and the RBF kernel function of SVM provide nonlinear classification ability. And CNN can also obtain linear and nonlinear features through convolution. These may be the reason why this study can obtain better classification results than the experiment of Oroian et al. in low-concentration adulteration.

## Conclusion

This study used Raman spectroscopy combined with machine learning algorithms to accurately detect the adulteration of low-concentration maltose syrup of Suichang native honey. And we used sensitivity, specificity and accuracy to evaluate CNN, PNN and SVM models. This study shows that Raman spectroscopy combined with machine learning algorithms can obtain extremely high accuracy in detecting low-concentration adulterated Suichang native honey. This method is a non-destructive, fast, efficient and highly accurate detection method for honey adulteration. We hope it can provide reference for relevant departments.

## Supplementary Information

Supplementary Figure S1.

Supplementary Figure S2.

Supplementary Figure S3.

Supplementary Figure S4.

Supplementary Figure S5.

Supplementary Table S1.

## Data Availability

Relevant data and spectra of five samples from both categories, pure honey and adulterated honey are available in the Supplementary file.
