# Supplementary figures and images for: Raman spectroscopy combined with machine learning algorithms to detect adulterated Suichang native honey
Source: Sci Rep. 2022 Mar 2;12:3456. doi: 10.1038/s41598-022-07222-3 (PMC8891316; doi:10.1038/s41598-022-07222-3)

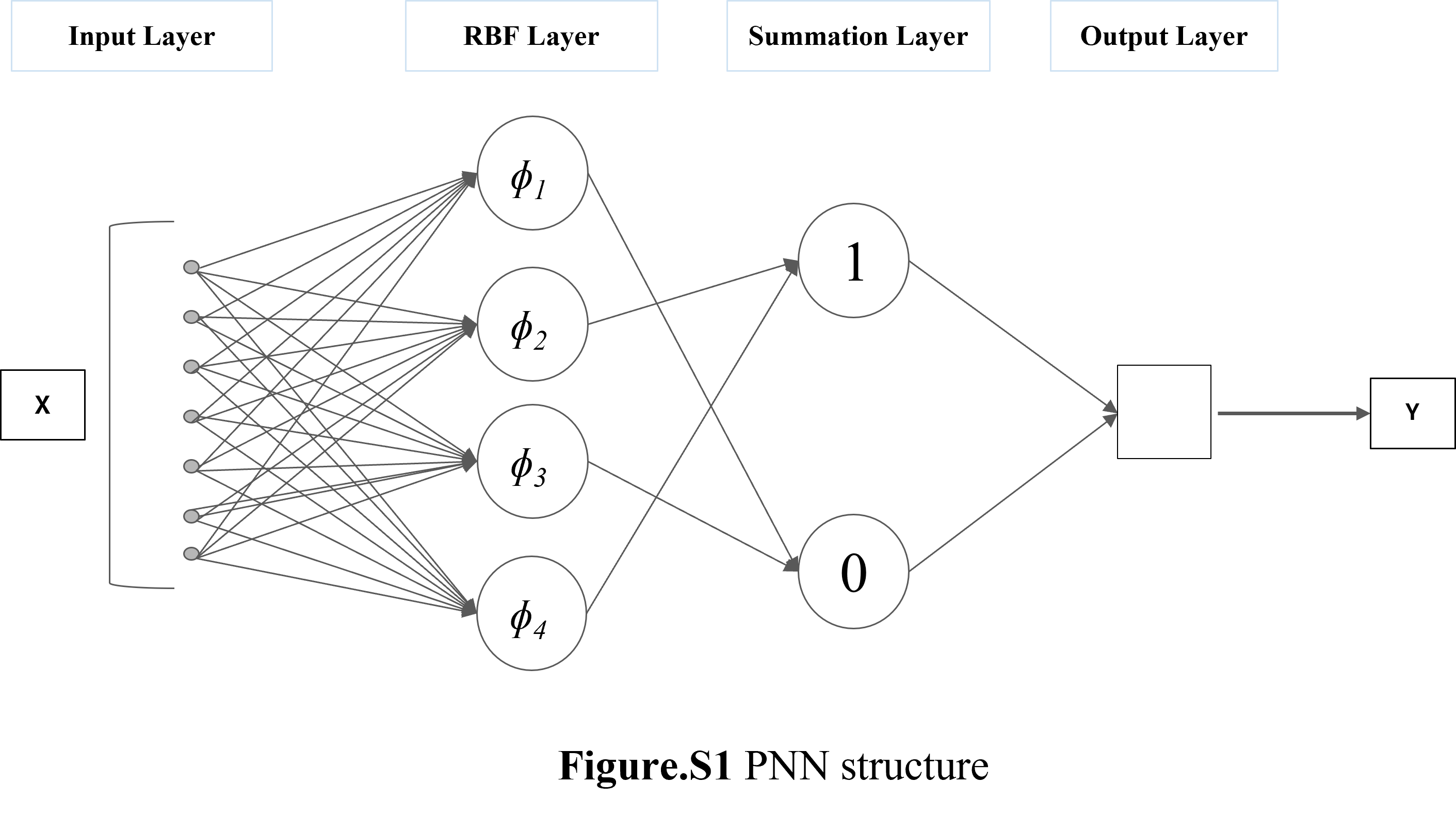

Supplement: Supplementary file 1 — Supplementary Figure S1. [file 41598_2022_7222_MOESM1_ESM.tif]

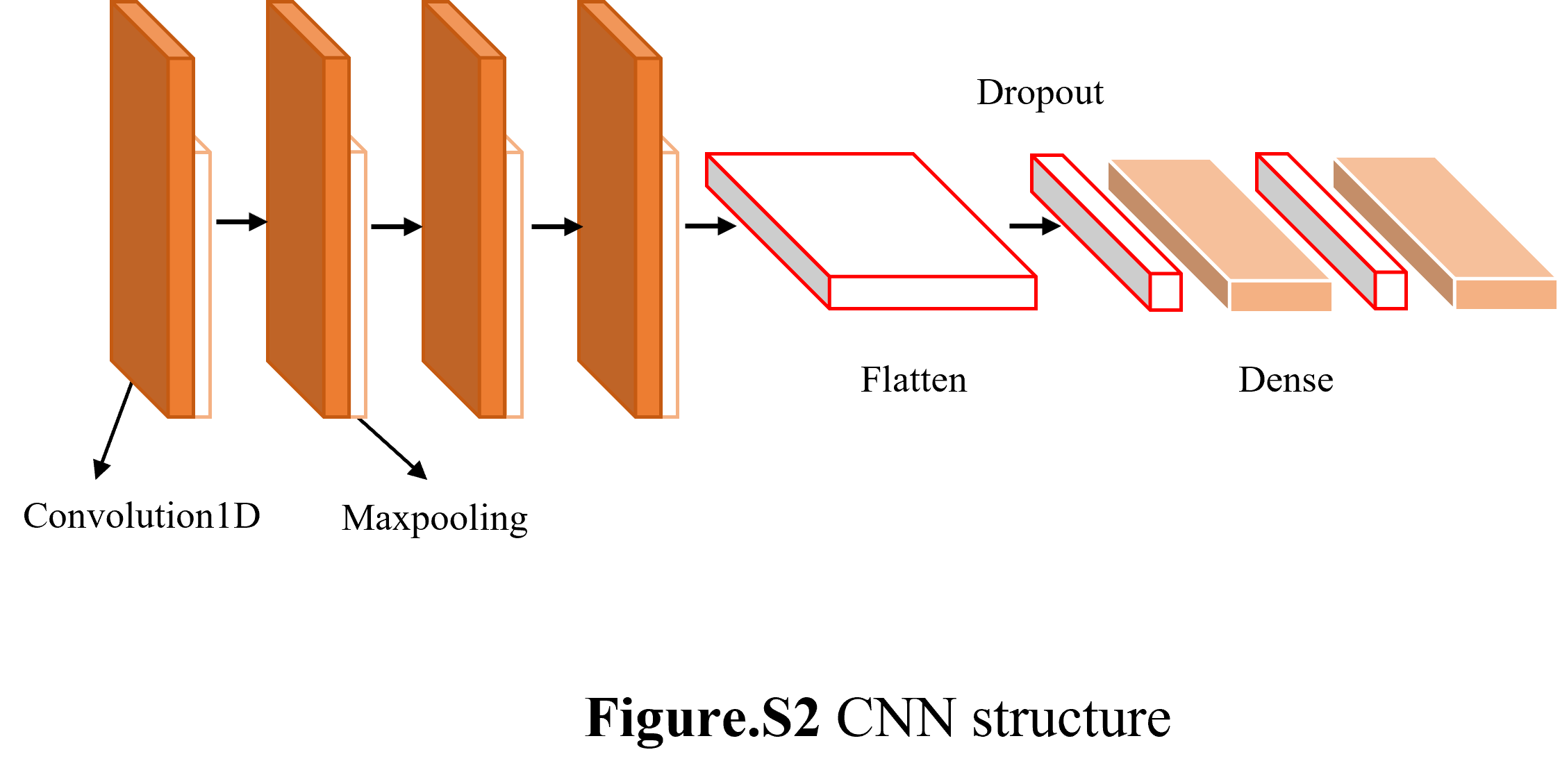

Supplement: Supplementary file 2 — Supplementary Figure S2. [file 41598_2022_7222_MOESM2_ESM.tif]

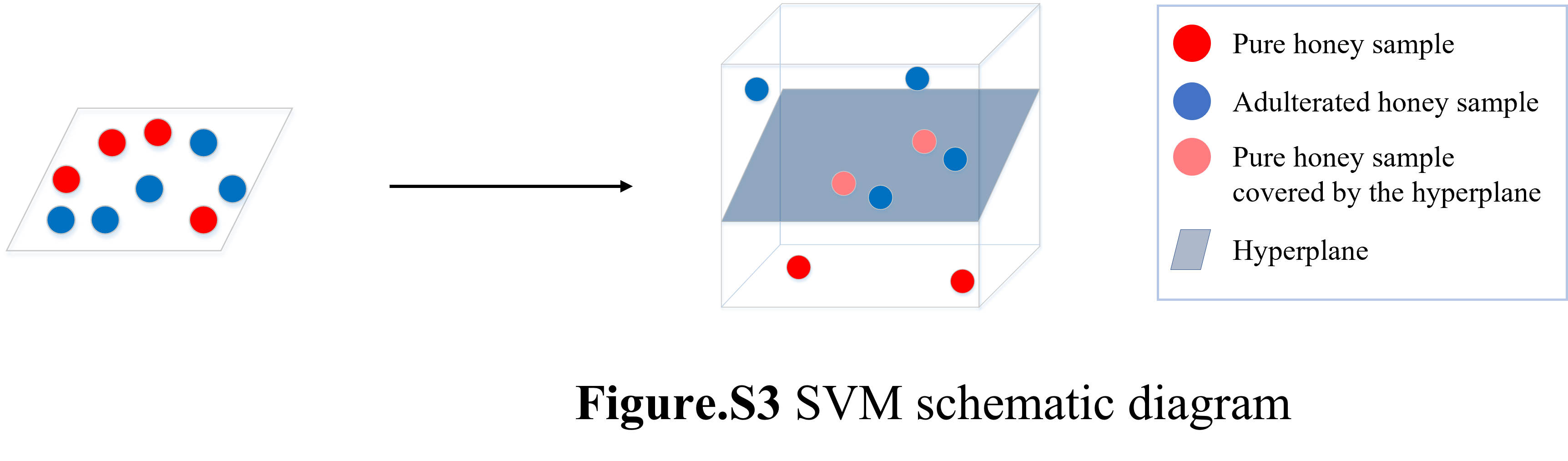

Supplement: Supplementary file 3 — Supplementary Figure S3. [file 41598_2022_7222_MOESM3_ESM.tif]

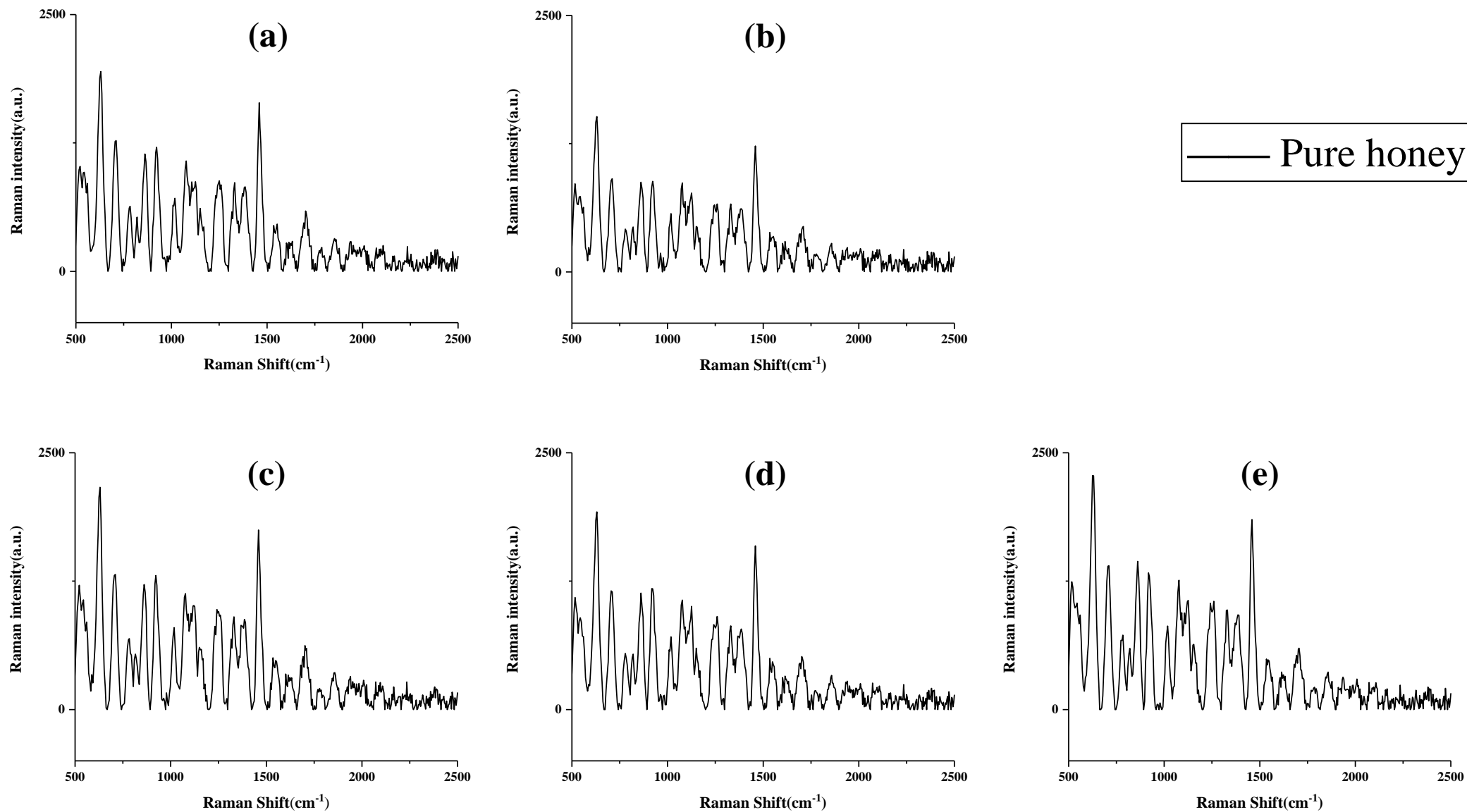

**Figure.S4** Raman spectra of five pure honey samples

Supplement: Supplementary file 4 — Supplementary Figure S4. [file 41598_2022_7222_MOESM4_ESM.pdf]

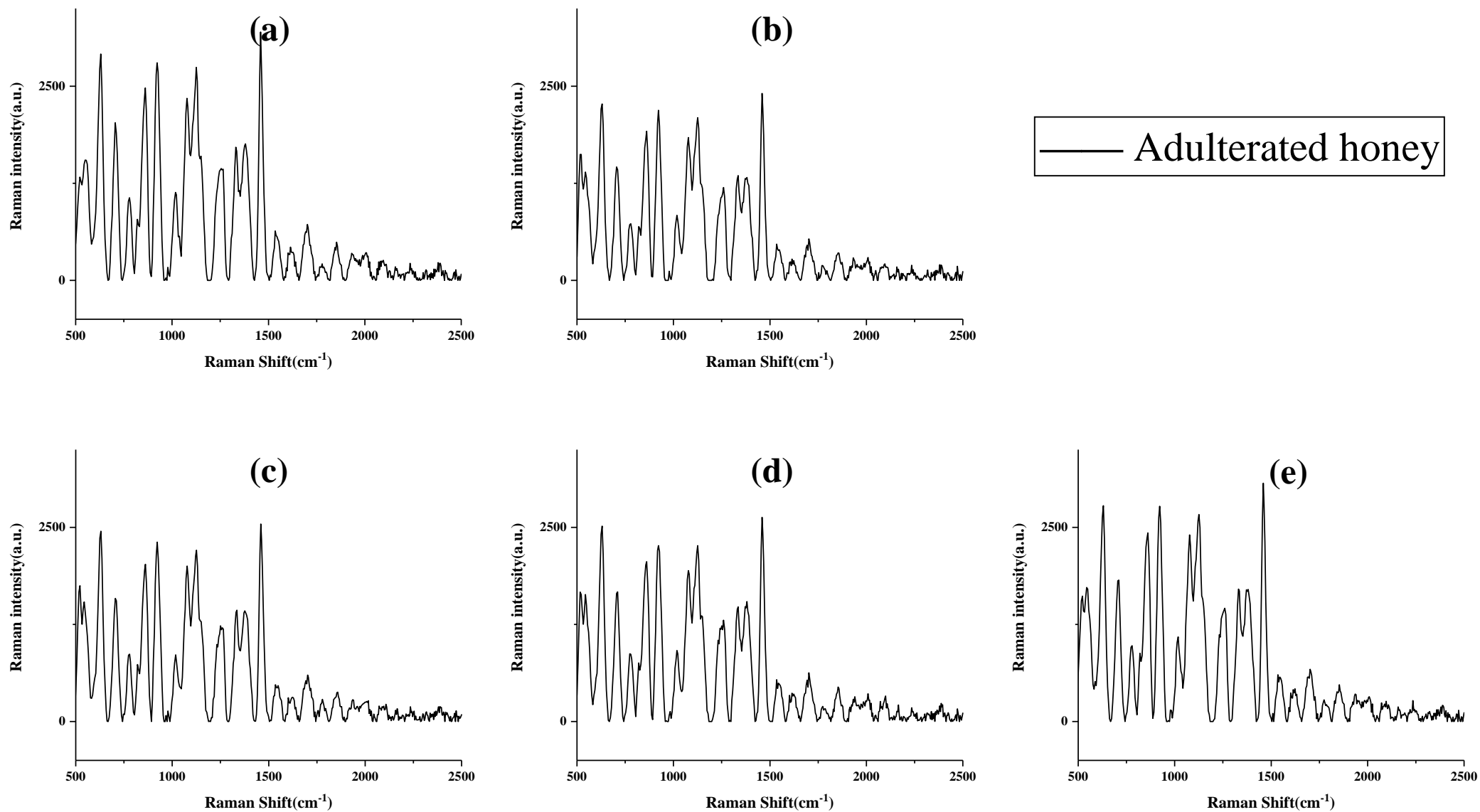

**Figure.S5** Raman spectra of five adulterated honey samples

Supplement: Supplementary file 5 — Supplementary Figure S5. [file 41598_2022_7222_MOESM5_ESM.pdf]
